# Overexpression of *BnaA10.WRKY75* Decreases Cadmium and Salt Tolerance via Increasing ROS Accumulation in *Arabidopsis* and *Brassica napus* L.

**DOI:** 10.3390/ijms25148002

**Published:** 2024-07-22

**Authors:** Xiaoke Ping, Qianjun Ye, Mei Yan, Jia Wang, Taiyuan Zhang, Sheng Chen, Kadambot H. M. Siddique, Wallace A. Cowling, Jiana Li, Liezhao Liu

**Affiliations:** 1 College of Agronomy and Biotechnology, Southwest University, Chongqing 400715, China; 2 The UWA Institute of Agriculture, The University of Western Australia, Perth, WA 6000, Australia

**Keywords:** *BnaA10.WRKY75*, cadmium stress, salt stress, *CAT2*, *Brassica napus* L.

## Abstract

Soil is indispensable for agricultural production but has been seriously polluted by cadmium and salt in recent years. Many crops are suffering from this, including rapeseed, the third largest global oilseed crop. However, genes simultaneously related to both cadmium and salt stress have not been extensively reported yet. In this study, *BnaA10.WRKY75* was screened from previous RNA-seq data related to cadmium and salt stress and further analyses including sequence comparison, GUS staining, transformation and qRT-PCR were conducted to confirm its function. GUS staining and qRT-PCR results indicated *BnaA10.WRKY75* was induced by CdCl_2_ and NaCl treatment. Sequence analysis suggested BnaA10.WRKY75 belongs to Group IIc of the WRKY gene family and transient expression assay showed it was a nuclear localized transcription factor. *BnaA10.WRKY75*-overexpressing *Arabidopsis* and rapeseed plants accumulated more H_2_O_2_ and O_2_^−^ and were more sensitive to CdCl_2_ and NaCl treatment compared with untransformed plants, which may be caused by the downregulation of *BnaC03.CAT2*. Our study reported that *BnaA10.WRKY75* increases sensitivity to cadmium and salt stress by disrupting the balance of reactive oxygen species both in *Arabidopsis* and rapeseed. The results support the further understanding of the mechanisms underlying cadmium and salt tolerance and provide *BnaA10.WRKY75* as a valuable gene for rapeseed abiotic stress breeding.

## 1. Introduction

As an important oil and economic crop, rapeseed (*Brassica napus* L.) is grown worldwide and is mainly used for human food, and the remaining meal is used in animal feed. With the development of modern industry, however, many plants are suffering from excessive cadmium in the soil, including rapeseed. Excessive cadmium not only reduces crop yields by disrupting multiple physiological processes but also enters the human food chain via plants, since it is accumulated in plant tissue such as seeds or leaves which are used in human food and animal feed [1,2]. For roots, cadmium toxicity inhibits the lateral root formation and root respiration. For leaves, cadmium will decrease carbon fixation efficiency and chlorophyll content, thereby resulting in reduced yield. Studies have also shown that cadmium will induce osmatic stress by regulating stomatal density and conductance and leaf relative water content [3,4]. Soil salinization is another challenge in agricultural production, which decreases the yield and reduces the arable land area in the world. High salt content in soil limits the absorption of water and nutrients, leading to primary stresses such as osmotic stress and ionic stress, which further disrupt many basic balances of plants [5,6]. More importantly, plants suffering from cadmium and salt stress exhibit similar phenotypes including more ROS accumulation and lower photosynthetic efficiency, which means that plant tolerance to both stresses can be enhanced by altering a single gene [7,8].

Some WRKY transcription factors that regulate genes related to cadmium and salt stress have been identified. GmWRKY142 negatively regulated Cd^2+^ uptake in cotton by upregulating the expression of *GmCDT1* [9]. Recent findings indicated that ZmWRKY64 upregulated *ZmSRG7* and maintained ROS homeostasis to mitigate cadmium toxicity in maize [10]. AtWRKY13 and AtWRKY45 positively regulated cadmium tolerance through regulating *AtPDR8* and *AtPCS1/2*, respectively [11,12]. AtWRKY12 increased cadmium accumulation by reducing the expression abundance of *AtGSH1* and plants overexpressing *AtWRKY12* showed decreased cadmium tolerance [13]. For salt stress, *PeWRKY41* is a positive regulator and transgenic poplar plants that overexpress *PeWRKY41* showed increased salt tolerance [14]. In *Arabidopsis*, both *AtWRKY25* and *AtWRKY33* play positive roles in resistance to salt stress [15]. Likewise, both *OsWRKY45* and *OsWRKY72* are positive regulatory factors for salt stress tolerance in rice [16,17]. Transgenic tobacco plants overexpressing *DgWRKY3* accumulated less H_2_O_2_ and malondialdehyde (MDA) and performed better under NaCl treatment than non-transformed plants [18]. The function of *WRKY75* in cadmium and salt toxicity tolerance has been reported in many species. Overexpression of *PyWRKY75* is beneficial for increasing the cadmium tolerance of poplar [19]. *AtWRKY75* and *AhWRKY75* have a positive effect on salt tolerance in *Arabidopsis* and peanuts, respectively [20,21]. On the contrary, knock-down lines of *PagWRKY75* have been shown to exhibit stronger tolerance to salt and transgenic poplar plants overexpressing *PagWRKY75* exhibited salt-sensitive phenotypes [22].

There are several studies aimed at screening genes associated with cadmium and salt stress tolerance in rapeseed [23,24,25], but limited studies have attempted to determine whether *BnaWRKY75s* respond to cadmium and salinity and there is no clear evidence to date to support a role of *BnaA10.WRKY75* in stress tolerance or sensitivity in rapeseed.

In this study, *BnaA10.WRKY75* was screened from previous transcriptome data based on the expression level change upon cadmium and salt stress. GUS staining and qRT-PCR experiments were undertaken and confirmed that *BnaA10.WRKY75* is responsive to these two types of stress. To investigate further its potential role in stress response, we generated *BnaA10.WRKY75*-overexpressing plants and evaluated their sensitivity to cadmium and salt stress, and also measured the ROS accumulated in such plants. The study aims to clarify the function of *BnaA10.WRKY75*, and its potential role in genetic solutions for resistance breeding.

## 2. Results

### 2.1. BnaWRKY75s Are Cadmium Induced

**Figure 1:**
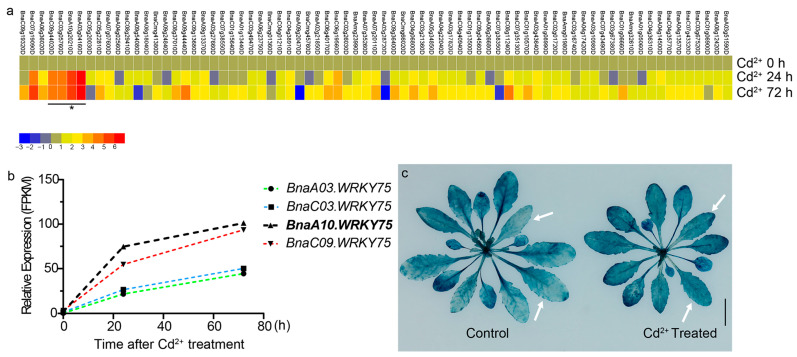
Response of *WRKY* transcription factors to cadmium stress. (**a**) Expression levels of 75 differently expressed *WRKY* transcription factors as revealed by RNA-seq. Expression levels were described by fold change and (Cd^2+^ 0 h) was used as the control. Four *BnaWRKY75s* and *BnaA10.WRKY75* were indicated by line and star, respectively. (**b**) Expression levels of four *BnaWRKY75s* under cadmium stress. (**c**) GUS staining results of *Arabidopsis* transgenic plants expressing *pBnaA10.WRKY75::GUS*. White arrows indicate the difference in GUS signal between cadmium treated and untreated plants. Bars: 1 cm.

We previously screened genes related to cadmium response in rapeseed seedlings [26]. Here, further analysis indicated that 75 WRKY transcription factors responded to cadmium treatment and all *BnaWRKY75s* on the *Darmor-bzh* reference genome were upregulated significantly (Figure 1a). Compared with the other three *BnaWRKY75s*, *BnaA10.WRKY75 (BnaA10g20210D)* showed the highest induced amount and was selected for further analysis (Figure 1b). The GUS staining results of transgenic *Arabidopsis* plants expressing *pBnaA10.WRKY75::GUS* also indicated that *BnaA10.WRKY75* was induced by cadmium treatment (Figure 1c). These results indicated that *BnaA10.WRKY75* is upregulated following cadmium treatment.

### 2.2. BnaWRKY75s Belong to Group IIc and Are Potentially Stress-Induced

**Figure 2:**
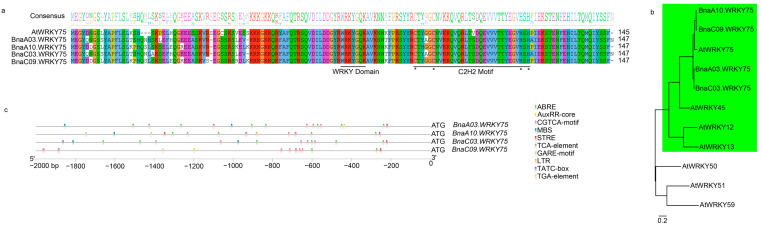
Protein sequences analysis and cis-element identification of four *BnaWRKY75s*. (**a**) Multiple sequences alignment. Two domains and representative amino acids were marked by line and star, respectively. (**b**) A phylogenetic tree including four BnaWRKY75s and 7 AtWRKY proteins from Group IIc. The green shading indicates the proteins that are closely related to BnaA10.WRKY75. (**c**) Genomic location of cis-element in *BnaWRKY75s* promoter.

Alignment of protein sequences showed that *BnaWRKY75s* contain two typical domains, namely the WRKY and C2H2 motif, and belong to the WRKY transcription factor family IIc subgroup (Figure 2a). A phylogenetic tree including seven AtWRKY proteins from Group IIc shown besides AtWRKY75; BnaWRKY75s also grouped with AtWRKY12, AtWRKY13, and AtWRKY45 (Figure 2b). All the three genes were proven to be associated with cadmium tolerance or sensitivity [11,12,13]. Promoter analysis indicated that *BnaWRKY75s* contain similar cis-element including stress- and plant hormone-related (Figure 2c). Gene clustering and promoter analysis also supported the idea that *BnaWRKY75s* are potentially involved in stress response.

### 2.3. BnaA10.WRKY75 Is Nuclear Localized and Highly Expressed in Senescent Leaves and Roots

**Figure 3:**
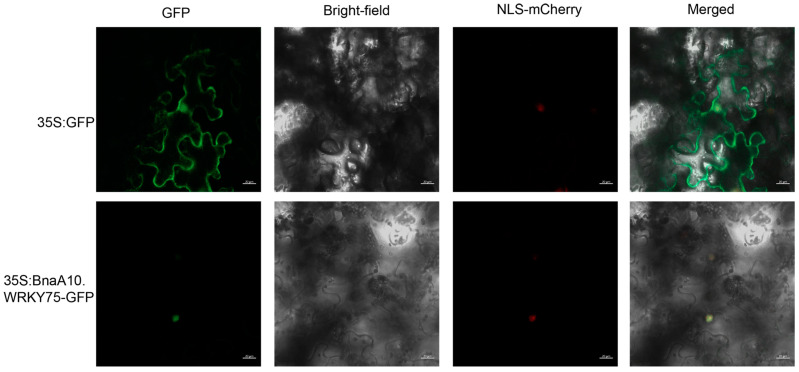
Localization of BnaA10.WRKY75 in tobacco epidermal cells.

**Figure 4:**
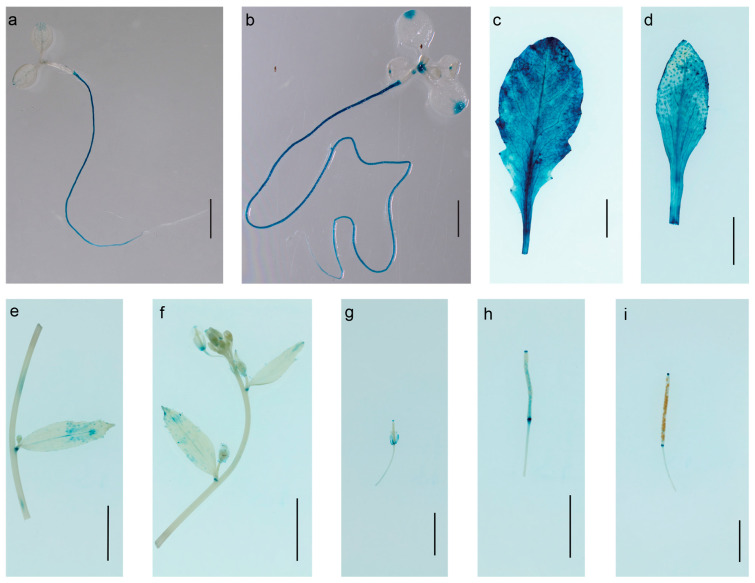
Expression of *BnaA10.WRKY75* in tissues detected by GUS staining: (**a**) 5 and (**b**) 14-day-old seedling; (**c**) 30-day-old leaf; (**d**) 10-day-old leaf; (**e**) stem; (**f**) flower; (**g**–**i**) silique at 1, 7 and 14 days after flowering. Bar: 1 cm.

A GFP fluorescence signal was detected in whole tobacco cells after injection of a positive control (PEGAD-GFP), but the fused protein of BnaA10.WRKY75-GFP was only located in the nucleus and co-localized with a nuclear marker, suggesting that BnaA10.WRKY75 is a nucleus-localized protein (Figure 3). Different stages and tissues of transgenic *Arabidopsis* plants expressing *pBnaA10.WRKY75::GUS* were stained and the results showed that *BnaA10.WRKY75* was highly expressed in roots of 5- and 14-day-old seedlings and senescent leaves (Figure 4a–c). The young leaves, flowers and siliques showed weaker signals; however, stems and seeds did not retain dye (Figure 4d–i). These results indicated that BnaA10.WRKY75 acts as a nucleus-localized transcription factor and mainly functions in roots and leaves.

### 2.4. Overexpression of BnaA10.WRKY75 Decreases Cadmium Tolerance in Arabidopsis and Rapeseed

**Figure 5:**
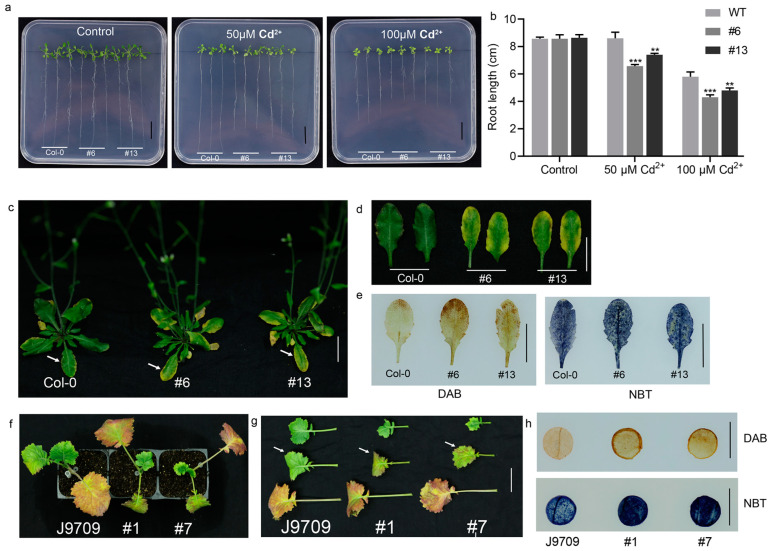
The effects of cadmium stress on wild-type and *BnaA10.WRKY75*-overexpressing plants. (**a**,**b**) Root length performance of seedlings grown on MS medium with or without CdCl_2_ added for three weeks. (**c**,**d**) Performance of leaves and (**e**) H_2_O_2_ and O_2_^−^ accumulation of plants irrigated by 500 μM CdCl_2_ solution for 7 d. White arrows in (**c**) indicate the difference in leaves between *BnaA10.WRKY75* overexpressing and Col-0 seedlings. (**f**,**g**) The performance of rapeseed seedlings irrigated by 1000 μM CdCl_2_ solution for 10 d and white arrows indicate the difference in leaves between *BnaA10.WRKY75* overexpressing and J9709 seedlings. (**h**) DAB and NBT staining results of rapeseed plants irrigated by 1000 μM CdCl_2_ solution. Values in (**b**) are the mean ± SD of three replications and differences in comparisons were revealed by student’s *t*-test. **, *p* < 0.01; ***, *p* < 0.001. Bars: (**a**,**c**–**e**,**h**) 1 cm; (**f**,**g**) 2 cm.

To verify the relationship between the expression level of *BnaA10.WRKY75* and cadmium tolerance, an overexpression vector was constructed and transformed into *Arabidopsis* cv. Col-0. Two homozygous transgenic lines with the highest expression level (#6 and #13) were selected for phenotypic analysis together with Col-0 (Figure S1a, Col-0, #6 and #13) seedlings growing on MS medium for two weeks showed similar root length. Under 100 μM CdCl_2_ treatment, however, the root length of line #6 and #13 was only 4.8 ± 0.17 cm and 4.3 ± 0.17 cm, respectively, while the root length of Col-0 was 5.8 ± 0.35 cm (Figure 5a,b). Overexpressed *Arabidopsis* seedlings grown in soil showed yellow leaves after being irrigated with CdCl_2_ solution for 7 d, while Col-0 seedlings were not affected under the same conditions and still displayed green leaves (Figure 5c,d). The results suggested that the expression level of *BnaA10.WRKY75* was negatively associated with cadmium tolerance. A more intense brown color formed in leaves of line #6 and #13 than that of Col-0 after diaminobenzidine (DAB) staining due to more H_2_O_2_ accumulation. Similarly, nitro blue tetrazolium (NBT) staining results showed O_2_^−^ content in leaves of line #6 and #13 was also higher than that of Col-0 (Figure 5e).

To further verify the negative correlation between the decreased cadmium tolerance of line #6 and #13 and ROS accumulation. *BnaA10.WRKY75*-overexpressing rapeseed lines were derived and line #1 and #7 with the highest expression level were chosen to perform phenotypic characterization and staining experiments together with their recipient line rapeseed cv. J9709 (Figure S1b and Figure 5f,g). The results showed that under cadmium treatment, leaves of J9709 were greener and had less accumulated H_2_O_2_ and O_2_^−^ compared with that of *BnaA10.WRKY75*-overexpressing rapeseed plants (Figure 5g,h). The phenotype identification results in *Arabidopsis* and rapeseed indicated that *BnaA10.WRKY75* negatively regulates cadmium tolerance.

### 2.5. BnaA10.WRKY75 Is Up-Regulated by Multiple Abiotic Stresses

**Figure 6:**
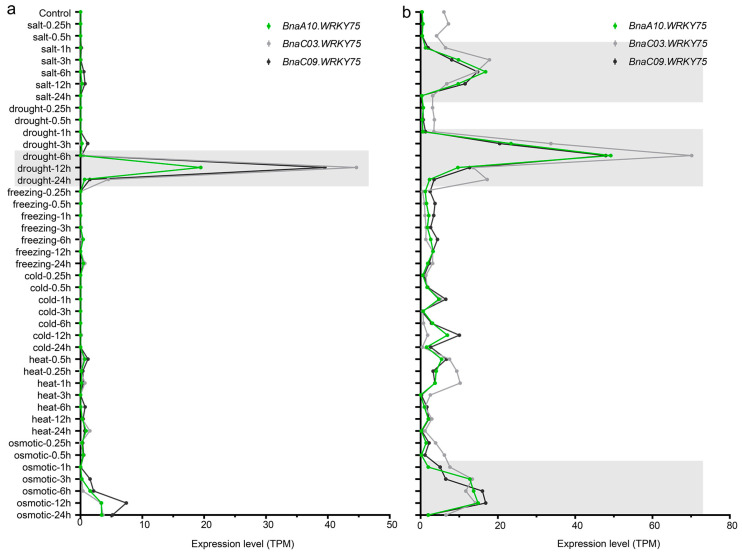
Response of *BnaWRKY75s* to stresses as obtained from BnIR database. (**a**,**b**) Expression patterns of *BnaWRKY75s* in leaves and roots, respectively. Gray shadings in (**a**,**b**) indicate significant upregulation of *BnaA10.WRKY75*.

Some WRKY transcription factors participated in regulating the tolerance to several other stresses [27]. To explore other stress responses that *BnaA10.WRKY75* may be involved in, the expression data of three of four *BnaWRKY75s* under multiple abiotic stresses were obtained from the BnIR database [28]. Analysis of *BnaA03.WRKY75* is not available because no *WRKY75* was identified on chromosome A03 in the ZS11 genome. *BnaA10.WRKY75*, *BnaC03.WRKY75* and *BnaC09.WRKY75* showed similar expression patterns, obviously induced by drought in leaves and by drought, salt and osmotic stress in roots (Figure 6).

**Figure 7:**
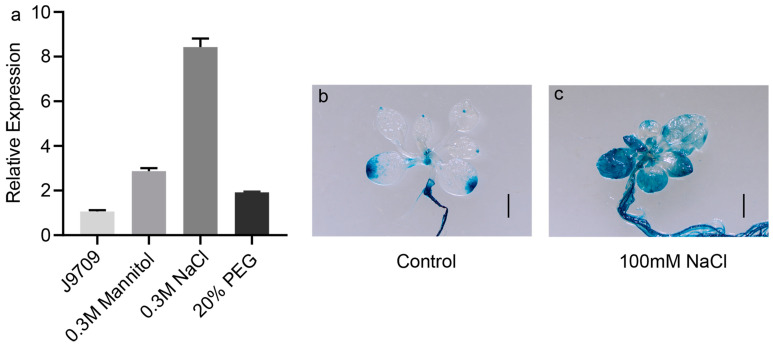
Response of *BnaA10.WRKY75* to three types of abiotic stress. (**a**) qRT-PCR result in rapeseed cv. J9709; (**b**,**c**) GUS staining results of transgenic *Arabidopsis* plants expressing *pBnaA10.WRKY75::GUS* under control and 100 mM NaCl treatments. Values in (**a**) are the mean ± SD of three replications. Bars: (**b**,**c**) 1 cm.

Mannitol, NaCl and polyethylene glycol (PEG) treatments were used to simulate osmotic, salt and drought stress, respectively. qRT-PCR was carried on rapeseed cv. J9709 and confirmed that *BnaA10.WRKY75* was upregulated by 1.92- to 8.43-fold under mannitol, salt and PEG treatment and the most significantly induced by salt (Figure 7a). Also, both leaves and roots of transgenic *Arabidopsis* plants expressing *pBnaA10.WRKY75::GUS* showed the enhanced GUS signal after NaCl treatment, which is consistent with the qRT-PCR result in rapeseed cv. J9709 and the expression data downloaded from the BnIR database (Figure 7b).

### 2.6. Overexpression of BnaA10.WRKY75 Decreases Salt Tolerance in Arabidopsis and Rapeseed

**Figure 8:**
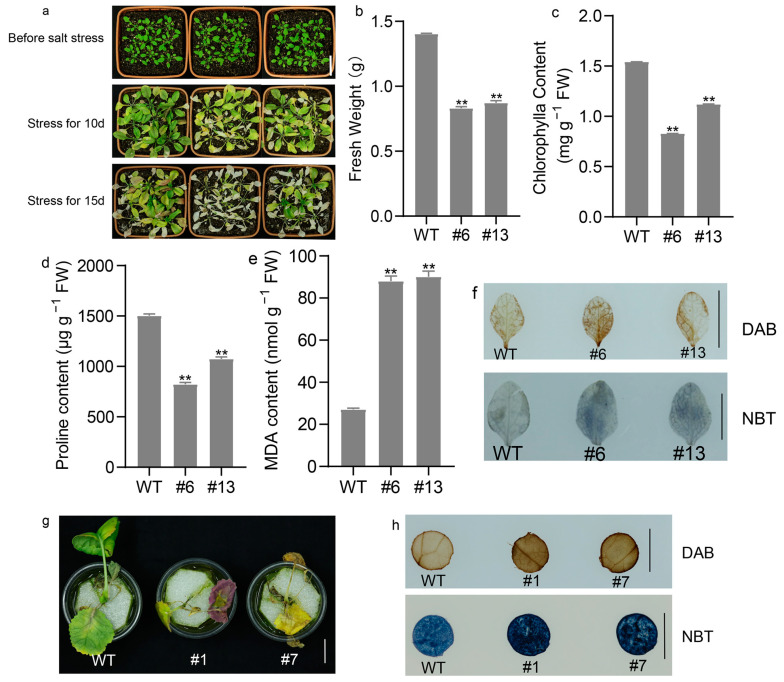
The effects of salt stress on wild-type and *BnaA10.WRKY75*-overexpressing plants. (**a**) Comparison of plant performance under salt treatment. (**b**–**e**) Differences in fresh weight, chlorophyll, proline and MDA content between Col-0 and transgenic *Arabidopsis* plants. (**f**) DAB and NBT staining revealed H_2_O_2_ and O_2_^−^ accumulation in leaves of *Arabidopsis* plants under salt treatment. (**g**) Performance of hydroponic rapeseed seedlings treated with salt solution for 10 d. (**h**) DAB and NBT staining revealed H_2_O_2_ and O_2_^−^ accumulation in leaves of rapeseed plants under salt treatment. Values in (**b**–**e**) are the mean ± SD of three replications and differences in comparisons were revealed by student’s *t*-test. **, *p* < 0.01. Bars: (**f**,**h**) 1 cm; (**a**,**g**) 2 cm.

The results in Section 2.5 guided us to explore whether *BnaA10.WRKY75* plays a role in altering salt tolerance. Upon NaCl treatment, Col-0 plants were less inhibited in growth and with higher fresh weight production and chlorophyll content (Figure 8a–c). The fresh weights of line #6 and #13 were 0.83 ± 0.01 g and 0.87 ± 0.01 g, respectively, which were significantly lower than that of Col-0 plants (1.4 ± 0.004 g). The chlorophyll content of Col-0 was 1.54 ± 0.009 mg g^−1^ FW, which was higher than those of #6 and #13 lines (0.83 ± 0.004 mg g^−1^ FW and 1.12 ± 0.008 mg g^−1^ FW, respectively) (Figure 8a–c). The content determination results of proline and MDA showed that Col-0 plants produced more proline but less MDA compared with transgenic *Arabidopsis* plants overexpressing *BnaA10.WRKY75* (Figure 8d,e). The proline content of Col-0 plant leaves was 1.40- to 1.82-fold higher than that of line #6 and #13, but the MDA content of Col-0 plant was 27.15 ± 0.53 nmol g^−1^ FW and the numbers of #6 and #13 lines were 88.02 ± 2.48 nmol g^−1^ FW and 90.11 ± 2.72 nmol g^−1^ FW, respectively (Figure 8d,e). Similar results to those in *Arabidopsis*, *BnaA10.WRKY75*-overexpressing rapeseed plants were more severely damaged by salt stress compared with J9709 (Figure 8g). The staining results of DAB and NBT demonstrated that leaves of *BnaA10.WRKY75*-overexpressing *Arabidopsis* and rapeseed plants contained more H_2_O_2_ and O_2_^−^ under salt stress (Figure 8f,h).

### 2.7. Identification of Genes Regulated by BnaA10.WRKY75

**Figure 9:**
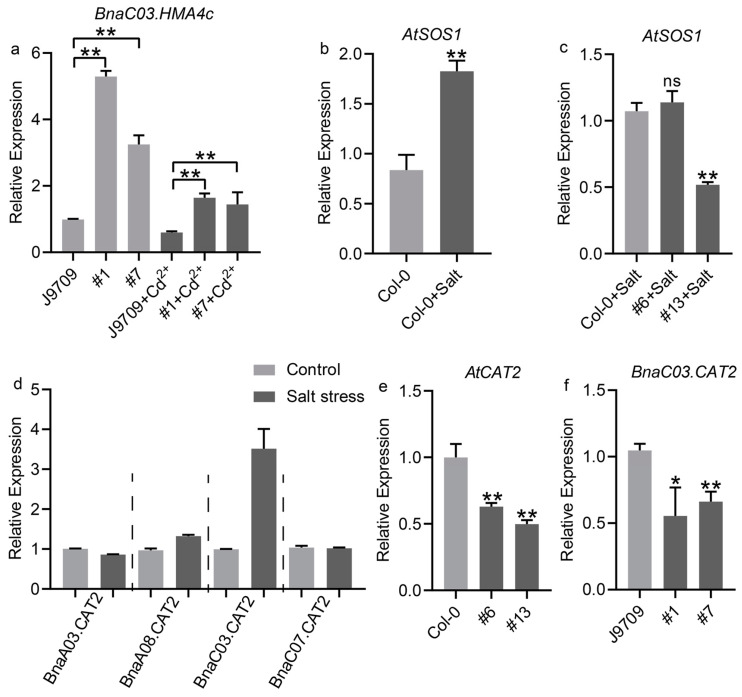
BnaA10.WRKY75 regulates the expression of genes related to cadmium and salt stress. (**a**) *BnaC03.HMA4c*, (**b**,**c**) *AtSOS1*, (**d**) *BnaCAT2s*, (**e**) *AtCAT2* and (**f**) *BnaC03.CAT2*. Values in (**a**–**f**) are the mean ± SD of three replications and differences in comparisons were revealed by student’s *t*-test. *, *p* < 0.05; **, *p* < 0.01; ns, no significance.

**Figure 10:**
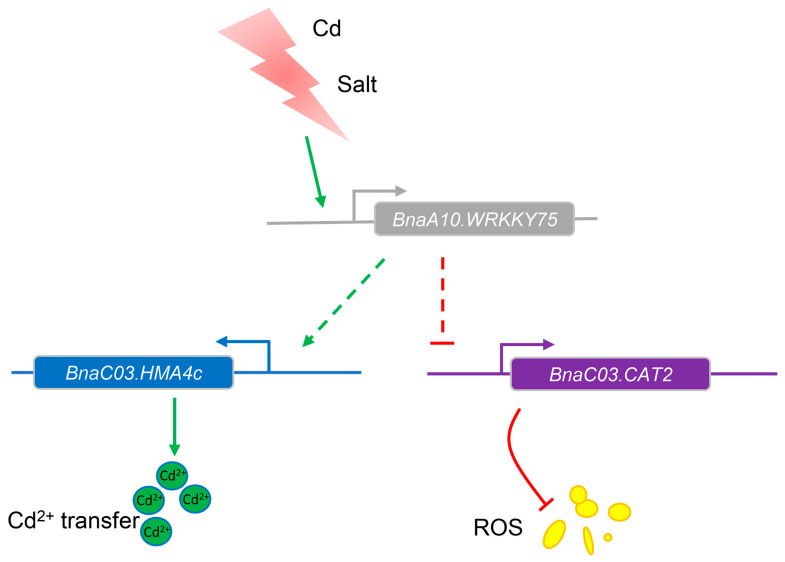
The role and working frame of *BnaA10.WRKY75* in response to cadmium and salt stress. Green lines mean promotion and red lines mean inhibition. Solid and dashed lines represent determined and undetermined regulatory relationships, respectively. *BnaA10.WRKY75* was induced by cadmium and salt stress then repressed *BnaC03.CAT2*, which is responsible for ROS scavenging. *BnaA10.WRKY75* also promotes the expression of *BnaC03.HMA4c* and increases Cd^2+^ transport.

*BnaC03.HMA4c*, a Cd^2+^ transport gene in rapeseed, was significantly upregulated (3.24- to 5.51-fold) in *BnaA10.WRKY75*-overexpressing rapeseed plants under control conditions (Figure 9a). After cadmium treatment, although its expression was decreased, it was still 2.4- to 2.7-fold higher than that of J9709 (Figure 9a). *AtSOS1*, a salt stress response gene, was highly induced by salt in Col-0 seedlings but the inducement was not further enhanced in *BnaA10.WRKY75*-overexpressing *Arabidopsis* seedlings and even down-regulated in line #13 (Figure 9b,c). The results indicated that *BnaA10.WRKY75* regulates salt tolerance in an *AtSOS1*-independent manner. ROS was accumulated in *BnaA10.WRKY75*-overexpressing plants under cadmium and salt treatment, and *CAT2* is responsible for scavenging ROS (Figure 5e,h and Figure 8f,h). There are four *BnaCAT2s* with relatively high expression levels in the ZS11 genome and *BnaC03.CAT2* showed the highest induced amount by salt treatment (Figure 9d). The expression abundance of *AtCAT2* and *BnaC03.CAT2* was decreased in *Arabidopsis* and rapeseed plants overexpressing *BnaA10.WRKY75*, respectively (Figure 9e,f). The expression patterns of these genes suggested that downregulation of *AtCAT2* and *BnaC03.CAT2* might be responsible for the accumulation of ROS, which in turn made *BnaA10.WRKY75*-overexpressing plants more sensitive to cadmium and salt stress. Regarding *BnaC03.HMA4c*, it seems to be only associated with the increased cadmium sensitivity in overexpressing plants (Figure 10).

## 3. Discussion

### 3.1. Functional Identification of BnaA10.WRKY75 in Rapeseed

The WRKY transcription factors function powerfully in plant development and stress response [29]. WRKY75, a member belonging to Group IIc of the WRKY transcription factor family, was widely involved in salt and cadmium tolerance across different species [19,20,21,22]. Although a systematical identification about the WRKY gene family in rapeseed was conducted recently [27], whether *BnaA10.WRKY75* participates in stress response and how it functions is largely unknown. In our previous research, genes responding to cadmium treatment were screened and *BnaA10.WRKY75* was included [26]. Continuing in this study, we created *BnaA10.WRKY75*-overexpressing *Arabidopsis* and rapeseed plants and confirmed that *BnaA10.WRKY75* overexpression increases cadmium sensitivity (Figure 5). Similar results were found under salt stress (Figure 8). As far as we know, *BnaA10.WRKY75* is the first WRKY transcription factor involved in cadmium and salt stress responses in rapeseed confirmed by transgenic plants. The results of this study enrich the research reports on the function of WRKY75 in abiotic stress, which not only makes up for the lack of functional verification in previous studies, but also provides feasibility for using *BnaA10.WRKY75* for resistance breeding in the future.

### 3.2. BnaA10.WRKY75 May Be a Basic Stress Response Factor

The WRKY transcription factors can generally be classified into Group I-III and Group II consists of five subgroups, IIa-e [30]. *AtWRKY75* was clustered into Group IIc with other 16 *AtWRKY* members, including genes that have been proven to function under stress conditions, such as *AtWRKY8*, *AtWRKY43*, *AtWRKY57* and *AtWRKY71*, which function in salt tolerance or sensitivity [31,32,33,34], *AtWRKY28* and *AtWRKY48* which function in biotic stress tolerance or sensitivity [35,36], and *AtWRKY12*, *AtWRKY13* and *AtWRKY45* which function in cadmium tolerance or sensitivity [11,12,13]. Genes with similar protein sequences and structures would be clustered into the same group and this also means that they may have similar functions, so these studies related to other members in Group IIc suggested that *BnaA10.WRKY75* may also function in expressing stress tolerance or sensitivity. RNA-seq data showed that *BnaA10.WRKY75* was regulated to varying degrees by various stresses (Figure 6a,b). Cis-element analysis also indicated that *BnaA10.WRKY75* was potentially induced or indirectly regulated by stress (Figure 2c). ROS homeostasis is crucial for plants and CAT is a key enzyme for scavenging excessive ROS induced by stress. In our study, *AtCAT2* and *BnaC03.CAT2* was downregulated in overexpressing *Arabidopsis* and rapeseed plants, respectively (Figure 9e,f), which hinted that *BnaA10.WRKY75* may serve as a basic stress response factor by sensing stress signals and downregulating *CAT2*.

### 3.3. Functional Differentiation of WRKY75 among Species

As described above, *WRKY75s* participated in similar stresses among species, but functional differentiation is also obvious. Overexpression of *PagWRKY75* in poplar decreased salt and osmotic tolerance [22], which is consistent with the function of *BnaA10.WRKY75* we report in the present study. *AhWRKY75* in peanuts and *AtWRKY75* in *Arabidopsis* both positively regulated salt tolerance and *AtWRKY75* acted by activating *AtSOS1* [20,21]. Compared to Col-0 plants, however, *BnaA10.WRKY75*-overexpressing *Arabidopsis* plants did not have an increased expression of *AtSOS1* under salt stress in this study (Figure 9c), which indicates that unlike *AtWRKY75*, *BnaA10.WRKY75* may not be active in regulating *AtSOS1*. Increasing the expression level of *PyWRKY75* can enhance the cadmium tolerance in poplar [19], while highly expressed *BnaA10.WRKY75* has the opposite effect in this study (Figure 5). Sequences alignment showed *PagWRKY75*, *AhWRKY75*, *PyWRKY75*, *AtWRKY75* and *BnaA10.WRKY75* all belong to Group IIc and have the same WRKY domain (WRKYGQK) and C2H2 finger motif (Cx_4_Cx_23_HxH) (Figure 2a), so amino acids that differ in other positions may determine the function of these WRKY75 proteins or different genes targeted by the WRKY75 transcription factor in different species. Further research is required to clarify this issue, but genetic functional differentiation among species is apparent.

## 4. Materials and Methods

### 4.1. Plant Materials and Stress Treatment

*Arabidopsis* and rapeseed seeds were sown on 1/2 MS phytoagar medium after being surface sterilized with 75% alcohol for 5 min. Seedlings that were grown for 10 days (d) were transplanted to soil. All seedlings grew in a chamber with conditions set at 22 ± 2 °C, 15,000 lx and 16 h photoperiod.

For phenotypic analysis, DAB and NBT staining of *Arabidopsis*, 300 mM NaCl and 500 μM CdCl_2_ solutions were used to irrigate 2-week-old soil cultured seedlings for 7 d, respectively. Three-week-old seedlings grown on 1/2 MS phytoagar medium containing 0, 50 and 100 μM CdCl_2_ were used to measure root length.

For phenotypic analysis, DAB and NBT staining of rapeseed, 10-day-old (BBCH growth stage 11) and 2-week-old (BBCH growth stage 12) rapeseed seedlings were hydroponically cultured with 100 mM NaCl solution and irrigated by 1 mM CdCl_2_ solution for 10 d, respectively.

For expression pattern analysis of *BnaA10.WRKY75*, 3-week-old (BBCH growth stage 13) hydroponic J9709 seedlings cultured in 100 mL of 1/2 strength MS solution were treated with 300 mM mannitol, 300 mM NaCl and 20% PEG for 2 h and the third true leaves were used to extract RNA. The seedlings were fixed by a 5 cm diameter round foam to maintain vertical growth in the whole process. Tissues from normally growing transgenic plants expressing *pBnaA10.WRKY75::GUS* and four-week-old seedlings irrigated with 300 mM CdCl_2_ solution for 3 d and seedlings grown on 1/2 MS phytoagar medium containing 100 mM NaCl for 20 d were stained by GUS solution.

For expression analysis of genes regulated by *BnaA10.WRKY75*, 10-day-old *Arabidopsis* and rapeseed seedlings (BBCH growth stage 11) grown on blank 1/2 MS phytoagar medium or containing 100 mM NaCl or 100 μM CdCl_2_ were stored in liquid nitrogen until used.

### 4.2. Sequence Alignment and Cis-Element Prediction

The protein sequences of AtWRKYs and BnaWRKY75s were respectively downloaded from TAIR (http://www.arabidopsis.org, accessed on 5 February 2024) and *Darmor-bzh* reference genome (http://www.genoscope.cns.fr/brassicanapus/, accessed on 5 February 2024). MEGA11 (Temple University, Philadelphia, PA, USA) software and the maximum likelihood method with default parameters were applied to construct a phylogenetic tree [37]. The cis-elements contained in the sequences of 2000 bp upstream of the initiation codons (ATG) were identified in the PlantCARE database (http://bioinformatics.psb.ugent.be/webtools/plantcare/html/, accessed on 5 February 2024) [38]. TBtools2.005 (South China Agricultural University, Guangzhou, China) was used to visualize the alignment results and locations of cis-element [39].

### 4.3. Plant Expression Vector Construction and Plant Transformation

The fragments of the *BnaA10.WRKY75* coding sequence and its promoter were amplified from the genome of rapeseed cv. ZS11 and inserted into DsRed and pCAMBIA1305.1 vectors, respectively. Primer sequences were supplied in Table S1. Vectors were transferred into *A. thaliana* cv. Col-0 and rapeseed cv. J9709 by *Agrobacterium* infection to obtain transgenic plants as reported [40,41]. The positive individuals were confirmed in every generation and a homozygous transformed progeny was used for experiments.

### 4.4. GUS Staining and Subcellular Localization

Homozygous transgenic seedlings expressing *pBnaA10.WRKY75::GUS* were stained by referring to the instructions in the GUS staining kit (Coolaber, Beijing, China). In brief, the tissues or whole seedlings were stained for 24 h then decolorized by 75% alcohol until there was no chlorophyll residue in the tissues.

The coding sequence of *BnaA10.WRKY75* was amplified and inserted into the PEGAD vector, forming a fusion protein with GFP for BnaA10.WRKY75 localization. Both PEGAD-GFP and PEGAD-BnaA10.WRKY75-GFP were infiltrated into tobacco leaves with a nuclear marker as mentioned in a previous study [42]. The pictures were obtained by a Zeiss LSM780 laser scanning confocal microscope (Carl Zeiss AG, Oberkochen, Germany).

### 4.5. Physiological Characters Measurement and DAB and NBT Staining

Chlorophyll content was determined as follows: 0.1 g sampled leaf was sheared and soaked in 10 mL acetone under dark environment until the tissue was white. The absorbance value of samples at 663 and 645 nm were measured and acetone was used as a blank. The content was calculated as (8.02 × A663 + 20.21 × A645) × V/(1000 × m), where A663 and A645 mean the absorbance value at 663 and 645 nm, V = 10 mL, m = 0.1 g.

Proline in leaf samples (0.1 g) was extracted with 5 ml of sulfosalicylic acid solution (3%) in boiling water for 10 min. After cooling to room temperature, the mixture was centrifuged at 3000 rpm/min for 10 min and 2 mL of the supernatant was moved into another centrifuge tube and 2 mL of ice-cold acetic acid and 2 mL of color reagent (2.5% acidic ninhydrin) were added. Afterward, the tubes were put into boiling water for 30 min and 4 mL of toluene was added after cooling to room temperature. The absorbance was measured at a wavelength of 520 nm and toluene was used as a blank. The content was calculated as (X × V1)/(V2 × m), where X means the proline content obtained based on absorbance values at 520 nm and standard curves, V1 = 5 mL, V2 = 2 mL, m = 0.1 g.

Leaf samples (0.1 g) were ground and homogenized in 1.8 mL of 10% trichloroacetic acid and centrifuged at 4000 rpm/min for 10 min, then 0.9 mL of supernatant was transferred into another centrifuge tube containing 0.9 mL 0.67% thiobarbituric acid. Afterward, the mixture was put in boiling water for 15 min and cooled to room temperature quickly. After centrifugation at 4000 rpm/min for 10 min, the absorbance of the supernatant at 450, 532 and 600 nm was determined. The content was calculated as [6.45 × (A532−A600) −0.56 × A450] × V3 × V1/(V2 × m), where A532, A600 and A450 mean the absorbance value at 532, 600 and 450 nm, V1 = 0.9 mL, V2 = 1.8 mL, V3 = 1.8 mL, m = 0.1 g. All absorbance values during the chlorophyll, proline and MDA content measurement process were determined by a spectrometer (UV-1800, Shimadzu Corporation, Kyoto, Japan). The third true leaves were incubated in 1 mg/mL DAB or 0.2% NBT staining solution for 8 h under dark conditions and decolorized in 75% alcohol until there was no chlorophyll residue in the leaves.

### 4.6. qRT-PCR and Spatiotemporal Expression Analysis under Stress

The concentrations and integrity of total RNA extracted using Trizol reagent (Vazyme, Nanjing, China) were measured by NanoDrop 2000 (Thermo Fisher Scientific, Worcester, MA, USA) and electrophoresis, respectively. Reverse transcription and qRT-PCR assay were performed referring to the directions of Hifair^TM^ III 1st Stand cDNA Synthesis SuperMix kit (YeaSen, Shanghai, China) and Hieff qPCR SYBR Green Master Mix kit (YeaSen, Shanghai, China), respectively. The primers for the qRT-PCR assay were designed based on the *Darmor-bzh* reference genome and primer sequences were supplied in Table S1. *AtActin2* and *BnaActin7* were used as the reference gene in *Arabidopsis* and rapeseed, respectively, and expression data were quantified using 2^−∆∆Ct^ method.

Expression data of *BnaA10.WRKY75*, *BnaC03.WRKY75* and *BnaC09.WRKY75* under multiple stresses were obtained from BnIR database (https://yanglab.hzau.edu.cn/BnIR, accessed on 8 February 2024) and represented by TPM (Transcript per million) value [28].

## 5. Conclusions

Continuing our previous research, we selected *BnaA10.WRKY75* for further study and generated overexpressed transgenic *A. thaliana* and rapeseed lines. RNA-seq, qRT-PCR and GUS staining experiments revealed that *BnaA10.WRKY75* was upregulated by multiple stresses, especially cadmium and salt. Gene expression studies indicated that BnaA10.WRKY75 is a nuclear localization transcription factor and is significantly expressed in leaves and roots. Related gene expression analysis suggested downregulation of *CAT2* leading to ROS accumulation in *BnaA10.WRKY75*-overexpressing plants and this was associated with increased sensitivity to cadmium and salt stress. The results of our research indicate that *BnaA10.WRKY75* is a target gene in future research for knocking out gene expression to potentially improve stress tolerance.

## Data Availability

All figures and data are included in the manuscript and Supplementary Materials.

## Supplementary Materials

The following supporting information can be downloaded at: https://www.mdpi.com/article/10.3390/ijms25148002/s1.

## References

[1] Chandra R., Kang H. (2016). Mixed heavy metal stress on photosynthesis, transpiration rate, and chlorophyll content in poplar hybrids. For. Sci. Technol..

[2] John R., Ahmad P., Gadgil K., Sharma S. (2009). Cadmium and lead-induced changes in lipid peroxidation, antioxidative enzymes and metal accumulation in *Brassica juncea* L. at three different growth stages. Arch. Agron. Soil. Sci..

[3] Aslam M.M., Okal E.J., Waseem M. (2023). Cadmium toxicity impacts plant growth and plant remediation strategies. Plant Growth Regul..

[4] Haider F.U., Liqun C., Coulter J.A., Cheema S.A., Wu J., Zhang R., Wenjun M., Farooq M. (2021). Cadmium toxicity in plants: Impacts and remediation strategies. Ecotoxicol. Environ. Saf..

[5] Gong Z. (2021). Plant abiotic stress: New insights into the factors that activate and modulate plant responses. J. Integr. Plant Biol..

[6] Zhu J. (2002). Salt and drought stress signal transduction in plants. Annu. Rev. Plant Biol..

[7] Mohamed I.A., Shalby N., El-Badri A.M., Batool M., Wang C., Wang Z., Salah A., Rady M.M., Jie K., Wang B. (2022). RNA-seq analysis revealed key genes associated with salt tolerance in rapeseed germination through carbohydrate metabolism, hormone, and MAPK signaling pathways. Ind. Crops Prod..

[8] Abbas T., Rizwan M., Ali S., Adrees M., Zia-ur-Rehman M., Qayyum M.F., Ok Y.S., Murtaza G. (2018). Effect of biochar on alleviation of cadmium toxicity in wheat (*Triticum aestivum* L.) grown on Cd-contaminated saline soil. Environ. Sci. Pollut. Res..

[9] Cai Z., Xian P., Wang H., Lin R., Lian T., Cheng Y., Ma Q., Nian H. (2020). Transcription factor GmWRKY142 confers cadmium resistance by up-regulating the *cadmium tolerance 1-like* genes. Front. Plant Sci..

[10] Gu L., Hou Y., Sun Y., Chen X., Wang G., Wang H., Zhu B., Du X. (2024). The maize WRKY transcription factor ZmWRKY64 confers cadmium tolerance in *Arabidopsis* and maize (*Zea mays* L.). Plant Cell Rep..

[11] Li F., Deng Y., Liu Y., Mai C., Xu Y., Wu J., Zheng X., Liang C., Wang J. (2023). *Arabidopsis* transcription factor WRKY45 confers cadmium tolerance via activating *PCS1* and *PCS2* expression. J. Hazard. Mater..

[12] Sheng Y., Yan X., Huang Y., Han Y., Zhang C., Ren Y., Fan T., Xiao F., Liu Y., Cao S. (2019). The WRKY transcription factor, WRKY13, activates *PDR8* expression to positively regulate cadmium tolerance in *Arabidopsis*. Plant Cell Environ..

[13] Han Y., Fan T., Zhu X., Wu X., Ouyang J., Jiang L., Cao S. (2019). WRKY12 represses *GSH1* expression to negatively regulate cadmium tolerance in *Arabidopsis*. Plant Mol. Biol..

[14] Yu X., Lu B., Dong Y., Li Y., Yang M. (2022). Cloning and functional identification of *PeWRKY41* from Populus× euramericana. Ind. Crops Prod..

[15] Jiang Y., Deyholos M.K. (2009). Functional characterization of *Arabidopsis* NaCl-inducible *WRKY25* and *WRKY33* transcription factors in abiotic stresses. Plant Mol. Biol..

[16] Qiu Y., Yu D. (2009). Over-expression of the stress-induced *OsWRKY45* enhances disease resistance and drought tolerance in *Arabidopsis*. Environ. Exp. Bot..

[17] Song Y., Chen L., Zhang L., Yu D. (2010). Overexpression of *OsWRKY72* gene interferes in the abscisic acid signal and auxin transport pathway of *Arabidopsis*. J. Biosci..

[18] Liu Q., Zhong M., Li S., Pan Y., Jiang B., Jia Y., Zhang H. (2013). Overexpression of a chrysanthemum transcription factor gene, *DgWRKY3*, in tobacco enhances tolerance to salt stress. Plant Physiol. Biochem..

[19] Wu X., Chen Q., Chen L., Tian F., Chen X., Han C., Mi J., Lin X., Wan X., Jiang B. (2022). A WRKY transcription factor, *PyWRKY75*, enhanced cadmium accumulation and tolerance in poplar. Ecotoxicol. Environ. Saf..

[20] Lu K., Song R., Guo J., Zhang Y., Zuo J., Chen H., Liao C., Hu X., Ren F., Lu Y. (2023). CycC1; 1–WRKY75 complex-mediated transcriptional regulation of *SOS1* controls salt stress tolerance in *Arabidopsis*. Plant Cell.

[21] Zhu H., Jiang Y., Guo Y., Huang J., Zhou M., Tang Y., Sui J., Wang J., Qiao L. (2021). A novel salt inducible WRKY transcription factor gene, *AhWRKY75*, confers salt tolerance in transgenic peanut. Plant Physiol. Biochem..

[22] Zhao K., Zhang D., Lv K., Zhang X., Cheng Z., Li R., Zhou B., Jiang T. (2019). Functional characterization of poplar *WRKY75* in salt and osmotic tolerance. Plant Sci..

[23] Zhang F., Xiao X., Wu X. (2020). Physiological and molecular mechanism of cadmium (Cd) tolerance at initial growth stage in rapeseed (*Brassica napus* L.). Ecotoxicol. Environ. Saf..

[24] Chen L., Wan H., Yi B., Ma C., Tu J., Shen J. (2018). Genome-wide association study of cadmium accumulation at the seedling stage in rapeseed (*Brassica napus* L.). Front. Plant Sci..

[25] Wan H., Chen L., Guo J., Li Q., Wen J., Yi B., Ma C., Tu J., Fu T., Shen J. (2017). Genome-Wide Association Study Reveals the Genetic Architecture Underlying Salt Tolerance-Related Traits in Rapeseed (*Brassica napus* L.). Front. Plant Sci..

[26] Ding Y., Jian H., Wang T., Di F., Wang J., Li J., Liu L. (2018). Screening of candidate gene responses to cadmium stress by RNA sequencing in oilseed rape (*Brassica napus* L.). Environ. Sci. Pollut. Res..

[27] Chen H., Wang Y., Liu J., Zhao T., Yang C., Ding Q., Zhang Y., Mu J., Wang D. (2022). Identification of *WRKY* transcription factors responding to abiotic stresses in *Brassica napus* L. Planta.

[28] Yang Z., Wang S., Wei L., Huang Y., Liu D., Jia Y., Luo C., Lin Y., Liang C., Hu Y. (2023). BnIR: A multi-omics database with various tools for *Brassica napus* research and breeding. Mol. Plant.

[29] Song H., Cao Y., Zhao L., Zhang J., Li S. (2023). WRKY transcription factors: Understanding the functional divergence. Plant Sci..

[30] Eulgem T., Rushton P.J., Robatzek S., Somssich I.E. (2000). The *WRKY* superfamily of plant transcription factors. Trends Plant Sci..

[31] Hu Y., Chen L., Wang H., Zhang L., Wang F., Yu D. (2013). *Arabidopsis* transcription factor *WRKY8* functions antagonistically with its interacting partner VQ 9 to modulate salinity stress tolerance. Plant J..

[32] Geilen K., Heilmann M., Hillmer S., Böhmer M. (2017). WRKY43 regulates polyunsaturated fatty acid content and seed germination under unfavourable growth conditions. Sci. Rep..

[33] Yu Y., Wang L., Chen J., Liu Z., Park C.-M., Xiang F. (2018). WRKY71 acts antagonistically against salt-delayed flowering in *Arabidopsis thaliana*. Plant Cell Physiol..

[34] Tang W. (2018). Heterologous expression of transcription factor *AtWRKY57* alleviates salt stress-induced oxidative damage. Open Biotechnol. J..

[35] Wu L., Zhong G., Wang J., Li X., Song X., Yang Y. (2011). *Arabidopsis* WRKY28 transcription factor is required for resistance to necrotrophic pathogen, *Botrytis cinerea*. Afr. J. Microbiol. Res..

[36] Xing D., Lai Z., Zheng Z., Vinod K., Fan B., Chen Z. (2008). Stress-and pathogen-induced *Arabidopsis* WRKY48 is a transcriptional activator that represses plant basal defense. Mol. Plant.

[37] Tamura K., Stecher G., Kumar S. (2021). MEGA11: Molecular evolutionary genetics analysis version 11. Mol. Biol. Evol..

[38] Lescot M., Déhais P., Thijs G., Marchal K., Moreau Y., Van de Peer Y., Rouzé P., Rombauts S. (2002). PlantCARE, a database of plant cis-acting regulatory elements and a portal to tools for in silico analysis of promoter sequences. Nucleic Acids Res..

[39] Chen C., Wu Y., Li J., Wang X., Zeng Z., Xu J., Liu Y., Feng J., Chen H., He Y. (2023). TBtools-II: A “one for all, all for one” bioinformatics platform for biological big-data mining. Mol. Plant.

[40] Dun X., Zhou Z., Xia S., Wen J., Yi B., Shen J., Ma C., Tu J., Fu T. (2011). *BnaC. Tic40*, a plastid inner membrane translocon originating from *Brassica oleracea*, is essential for tapetal function and microspore development in *Brassica napus*. Plant J..

[41] Clough S.J., Bent A.F. (1998). Floral dip: A simplified method for Agrobacterium-mediated transformation of *Arabidopsis thaliana*. Plant J..

[42] Song H., Duan Z., Wang Z., Li Y., Wang Y., Li C., Mao W., Que Q., Chen X., Li P. (2022). Genome-wide identification, expression pattern and subcellular localization analysis of the *JAZ* gene family in *Toona ciliata*. Ind. Crops Prod..

