# Supplementary material for: Overexpression of BnaA10.WRKY75 Decreases Cadmium and Salt Tolerance via Increasing ROS Accumulation in Arabidopsis and Brassica napus L
Source: Int J Mol Sci. 2024 Jul 22;25(14):8002. doi: 10.3390/ijms25148002 (PMC11276826; doi:10.3390/ijms25148002)
Supplement: Supplementary file 1 [file ijms-25-08002-s001.zip › ijms-3114458-supplementary.pdf]

Supplementary Information

Journal Name: International Journal of Molecular Sciences

Manuscript Title: Overexpression of *BnaA10.WRKY75* decreases cadmium and salt tolerance via increasing ROS accumulation in *Arabidopsis* and *Brassica napus* L.

Authors: Xiaoke Ping, Qianjun Ye, Mei Yan, Jia Wang, Taiyuan Zhang, Sheng Chen, Kadambot H. M. Siddique, Wallace A. Cowling, Jiana Li, Liezhao Liu

Name, affiliation, and email of corresponding author:

Liezhao Liu

College of Agronomy and Biotechnology, Southwest University, Chongqing 400715, China.

liezhao@swu.edu.cn

Content: Table S1 and Figure S1

Table S1. List of primers used in the study

Primer name	Sequence (5'-3')
**Primers for constructing overexpression vector**	
BnaA10WRKY75 F	GGATCCATGGAAGGATATGATGATGGATCG
BnaA10WRKY75 R	AAGCTTAAAAGAAGAGTAGATTTGCATTTGGGTG
**Primers for constructing subcellular localization vector**	
subBnaA10WRKY75 F	GAATTCATGGAAGGATATGATGATGGATCG
subBnaA10WRKY75 R	AAGCTTAAAAGAAGAGTAGATTTGCATTTGGGTG
**Primers for constructing GUS reporter gene vector**	

Table S1 (*Continued*)

Primer name	Sequence (5'-3')
pBnaA10WRKY75 F	**GCG**AAGCTTGTAAGCGTCGTAGTAGCATCCTTG

pBnaA10WRKY75 R	**GTA**CCATGGGTTCCCAATTAACTCTGGAATC
**Primers for qRT-PCR**	
BnaHMA4c F	GGAGAGATAACTATGGCATGTGAGG
BnaHMA4c R	CTTCCTTCAAACCACAAACAGACAT
AtSOS1 F	GCAGGAAAGTGCATTGGTTCTTC
AtSOS1 R	AACCAATGCTGTTGCAGTCG
BnaA03.CAT2 F	GGAGCGTATTCCAGAGCGC
BnaA03.CAT2 R	TGGATGACAGTGGAGAAACGC
BnaA08.CAT2 F	GCTTCGCAGTCAAGTTCTACACT
BnaA08.CAT2 R	GTTTGGTTTAAGCGCGTGGAC
BnaC03.CAT2 F	TCACACCACCCTGAGAGCTTG
BnaC03.CAT2 R	GAGACTTGACTCCACACGTG
BnaC07.CAT2 F	CCGGCAAAGCTCACTACGTC
BnaC07.CAT2 R	CTCAGGCCAGGTCTTGGTC
AtCAT2 F	ACCTTTACACCAGAGAGGCAAG
AtCAT2 R	GACTTATCAGCCTGAGACCAGT
AtActin2 F	GGTAACATTGTGCTCAGTGGTGG
AtActin2 R	AACGACCTTAATCTTCATGCTGC
BnaActin 7 F	GGAAGCTCCTGGAATCCATGAGA
BnaActin 7 R	TCTTTGCTCATACGGTCAGCAATTCC

The bold and underlined nucleotides represent extra bases and recognition sites of restriction enzymes, respectively.

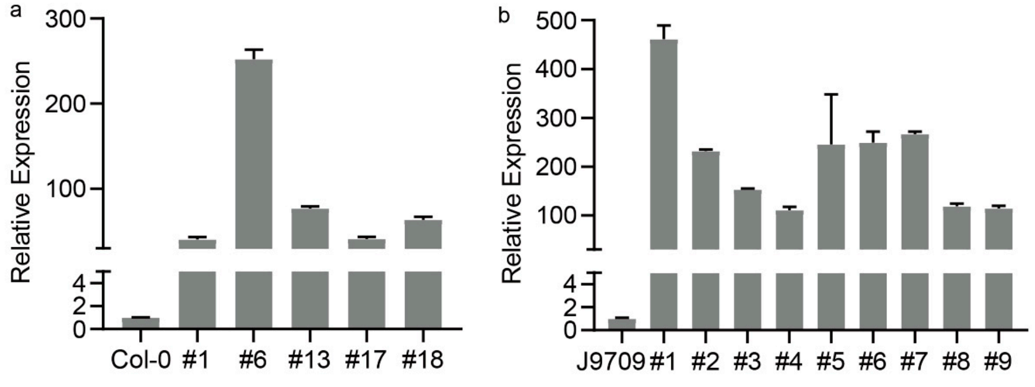

Figure S1. Overexpression level of *BnaA10.WRKY75* in transgenic **(a)** *Arabidopsis* and **(b)** rapeseed plants.
